# Household availability of foods from Brazilian biodiversity

**DOI:** 10.1590/0102-311XEN206222

**Published:** 2023-07-21

**Authors:** Marcos Anderson Lucas da Silva, Lucas Braga Rodrigues, Semíramis Martins Álvares Domene, Maria Laura da Costa Louzada

**Affiliations:** 1 Faculdade de Saúde Pública, Universidade de São Paulo, São Paulo, Brasil.; 2 Instituto de Cultura e Arte, Universidade Federal do Ceará, Fortaleza, Brasil.; 3 Instituto de Saúde e Sociedade, Universidade Federal de São Paulo, Santos, Brasil.

**Keywords:** Biodiversity, Biomes, Food System, Food Consumption, Sustainability, Sustainability Indicators, Biodiversidade, Biomas, Sistema Alimentar, Ingestão de Alimentos, Indicadores de Sustentabilidade, Biodiversidad, Biomas, Sistema Alimentario, Ingestión de Alimentos, Indicadores de Sostenibilidad

## Abstract

Food biodiversity is characterized by the diversity of foods that compose a local, regional, or national ecosystem. Brazil has 20% of all the planet’s biodiversity and the richest biomes in the world. Therefore, describing the participation of these foods in the Brazilian diet is relevant. Using a complex sample with data from 57,920 households collected by the Brazilian Institute of Geography and Statistics from 2017 to 2018, this study showed that, except for yerba mate, the availability of foods from Brazilian biodiversity is low, representing an average of 7.09g/per capita/day. Regarding biomes, the Caatinga had the highest availability of fruits (4.20g/per capita/day) while the Amazon had the highest availability of vegetables (1.52g/per capita/day). The results are unsatisfactory and lower than what is expected from a territory rich in biodiversity and a world-leading food system. A greater commitment is essential to promote actions that strengthen the consumption of these foods among Brazilians.

## Resumo

A biodiversidade alimentar é caracterizada pela diversidade alimentar que compõe um ecossistema local, regional ou nacional. O Brasil tem 20% de toda a biodiversidade do planeta e os biomas mais ricos do mundo. No entanto, é relevante descrever a participação desses alimentos na dieta dos brasileiros. Utilizando uma amostra complexa com dados de 57.920 domicílios, coletados pelo Instituto Brasileiro de Geografia e Estatística de 2017 a 2018, observou-se que, com exceção da erva-mate, a disponibilidade de alimentos provenientes da biodiversidade é baixa, representando uma média de 7,09g/*per capita*/dia. Por biomas, a Caatinga apresentou a maior disponibilidade de frutos (4,20g/*per capita*/dia), enquanto para legumes, a Amazônia se destacou (1,52g/*per capita*/dia). Os resultados são insatisfatórios e abaixo do que se espera de um território biodiverso e de um sistema alimentar que é destaque mundial. É necessário um maior comprometimento para a promoção de ações que fortaleçam o consumo desses alimentos entre brasileiros.

## Resumen

La biodiversidad alimentaria se caracteriza por la diversidad alimentaria que conforma un ecosistema local, regional o nacional. Brasil tiene el 20% de toda la biodiversidad del planeta y los biomas más ricos del mundo. Sin embargo, es relevante describir la participación de estos alimentos en la dieta de los brasileños. Utilizando una muestra compleja con datos de 57.920 domicilios, recopilados por el Instituto Brasileño de Geografía y Estadística de 2017 a 2018, se observó que, con excepción de la yerba mate, la disponibilidad de alimentos de la biodiversidad es baja, representando un promedio de 7,09g/per cápita/día. Por biomas, la Caatinga presentó la mayor disponibilidad de frutos (4,20g/per cápita/día), mientras que en relación a las legumbres, la Amazonía se destacó (1,52g/per cápita/día). Los resultados son insatisfactorios y por debajo de lo que se espera de un territorio rico en biodiversidad y un sistema alimentario mundialmente reconocido. Es necesario un mayor compromiso para la promoción de acciones que fortalezcan el consumo de estos alimentos entre los brasileños.

## Introduction

Food biodiversity is characterized by the diversity of species of plants, animals, and other organisms used in food in a local, regional, or national ecosystem ^1^. This definition is part of the conceptual umbrella of so-called healthy and sustainable food systems ^2^.

Responsible for 20% of all the planet’s biodiversity ^3^, Brazil has the largest number of species of flora ^4^ ^,^ ^5^ and the richest biomes in the planet, such as the Amazon, the Atlantic Forest, the Cerrado, the Caatinga, the Pantanal, and the Pampa ^6^. Therefore, Brazil has the potential to have one of the most biodiverse food systems in the world.

The strengthening of food systems involves the protection of biomes. This is one of the main strategies to combat environmental degradation and, at the same time, promote food sovereignty. Family farming is the main example of an action that directly contributes to the strengthening of these systems. Besides being the main source of food products available for consumption by the Brazilian population ^7^, it also seeks to balance the use of natural resources, actively participating in the transition process to ensure a sustainable agriculture and food system ^8^.

Consumer choice for biodiversity foods still largely depends on the availability of these foods and the conditions of access, which correspond to the demand, in a feedback loop ^9^. Production is reinforced by the strengthening of this demand, which leads to the protection of certain native species.

Few studies evaluate the use of biodiversity foods in the literature. Studies and official documents generally focus on the use of these foods for pharmaceutical products ^3^, in the culinary field ^6^, or on nutritional and sensory aspects ^10^ ^,^ ^11^ ^,^ ^12^. However, studies on the purchase of these foods in Brazilian households based on nationally representative data are still lacking.

Therefore, this study aims to describe the household availability of foods from Brazilian biodiversity and their relative participation in all Brazilian Federative Units and biomes from 2017 to 2018.

## Methods

### Data source and sampling

This study used household food purchase data from the *Brazilian Household Budgets Survey* (POF) conducted by the Brazilian Institute of Geography and Statistics (IBGE) from July 2017 to July 2018.

The survey used a complex sampling plan, grouped in two stages, involving the drawing of census sectors in the first stage and households in the second. The census sectors come from the IBGE master sample, grouped into strata of households with high geographic and socioeconomic homogeneity. The construction of the strata considered the geographic location, the area of residence (urban or rural for samples with national representation), and, within each geographic location, the spectrum of socioeconomic variation by the income of the head of household. Data collection from each survey was distributed over the four quarters of the year, incorporating the seasonal variety to which expenses are subject.

The estimates obtained in surveys with national samples are representative of the following domains: Brazil, the five macrorregions (North, Northeast, Central-West, Southeast, and South), area (urban or rural), the 27 Federative Units, the nine metropolitan areas, and the 27 Federative Units’ capitals. A detailed description of the survey sampling process is available in an IBGE publication ^13^.

### Data collection

The information used in this study refers to household food purchases during seven consecutive days, recorded by the household residents or an IBGE interviewer, considering monetary (cash, credit, and debit cards) and nonmonetary (donation, brought from work, exchange, own production) purchases. The aggregates of households generated in the sampling plan (strata) were used as the unit of study. From 2017 to 2018, the 57,920 households studied resulted in 575 strata with an average of 86.5 households (ranging from 16 to 524).

#### Identification and classification of biodiversity foods

Biodiversity foods were identified based on *Interministerial Ordinance n. 10*, of July 21, 2021 ^14^, which lists these foods for marketing purposes, either as fresh items or as products derived from these foods.

These foods were divided into groups of fruits (70) and vegetables (22), totaling 92 native foods. However, of these 92, only 38 (30 fruits - *abiu*, *açaí*, guava, *araticum*, *babassu*, *bacaba*, *bacuri*, Brazil nut, *biribá*, *amora*, *butia*, cashew, cocoa, *cupuaçu*, yellow mombin, genipap, *guarana*, guava, juçara palm, *mangaba*, passion fruit, peanut, peach palm, *pequi*, pineapple, pine nut, *pitanga*, *murici*, *tucumã*, and *umbu*; and 8 vegetables - cassava, *guariroba*, *gueroba*, *jambu*, *Major-gomes*, ora-pro-nobis, purslane and *taioba*) were found in the POF. These foods are native to Brazil, produced according to the biomes of each state. The aforementioned list of foods and the states where they are produced are available in the *Interministerial Ordinance n. 10/2021*.

To identify the foods reported in the POF with different names, but representing the same item (for example, tangerine, which in Brazil can be called “*tangerina*”, “*bergamota*”, among others), the Quality Index of the Coordination of Food and Nutrition Security ^15^, a tool of the Brazilian National Fund for Educational Development. This tool has descriptions of all foods with their variations and synonyms for each region of Brazil.

#### Classification of Brazilian biomes

Brazil has six biomes: Amazon, Atlantic Forest, Caatinga, Cerrado, Pantanal, and Pampa. All these biomes occupy a specific geographic space in the country, and it is relevant to consider the area that each biome occupies in each of the 27 Federative Units. Therefore, a partnership between the IBGE and the Brazilian Ministry of Environment resulted in a document entitled *Map of Biomes of Brazil - First Approximation* ^16^, which provides a table specifying the approximate percentage of the area occupied by each biome in the states. This percentage was used in this study to obtain a result closer to reality for the biomes, respecting the boundaries of each Federative Unit.

### Data analysis

#### Food availability

The per capita quantities of food were expressed in daily consumption values (g/per capita/day) after applying correction factors ^17^ to estimate the fraction available for consumption without inedible parts (peel or rotten parts).

A minimum cut-off line of 10% of the availability of food groups in each Federative Unit was created. Then, the amount consumed of each biodiversity group of fruits and vegetables was expressed based on mean (g/per capita/day) and relative (10%) availability in Brazilian Federative Units from 2017 to 2018.

#### Biomes

To obtain the availability of biodiversity food groups by biomes, the amount (g/per capita/day) available from each Brazilian state was used, considering the percentage of the area occupied by the biomes according to the *Map of Biomes of Brazil* ^16^. For example, in the case of the Pampa, a biome located only in Rio Grande do Sul, which represents about 63% of the territory of this Federative Unit and where the availability of biodiversity fruits was 100g, the analysis by biomes estimated that its availability of biodiversity fruits would be 63g/per capita/day.

Except for the Pampa, all other biomes occupy more than one Federative Unit. Equation 1 was used to make the respective estimations for each biome.

$$B=\frac{AAAO1+\ldots }{S}$$ (equation 1)

Where *B* is the biome and*AAAO1*is the amount available (g/per capita/day) in the area occupied by the biome; *S* is the number of Federative Units in which the biome is located; and *+...* represents the sum of the availability of the other states. The same formula was used for both biodiversity fruits and vegetables. Thus, these data express the availability (g/per capita/day) of fruits and vegetables from Brazilian biodiversity by biomes from 2017 to 2018.

Yerba mate was evaluated separately from other foods, as it is the only biodiversity food consumed exclusively by infusion (tea, *mate*, and *tererê*) in some Brazilian states. Thus, this study considered availability per Federative Unit. Detailed data for all results can be found in the Supplementary Material (https://cadernos.ensp.fiocruz.br/static//arquivo/suppl-e00206222_2112.pdf) with the respective 95% confidence intervals.

All analyses were performed using the Stata 14.0 statistical package (https://www.stata.com), and all data were reviewed by the author and coauthors of this study.

## Results

In Brazil, the total availability (g/per capita/day) of food corresponded to an average of 1,092g per day. Moreover, 39 biodiversity foods represented, on average, only 7.09g of this total: 5.89g from fruits and 1.20g from vegetables.

**Figure 1:**
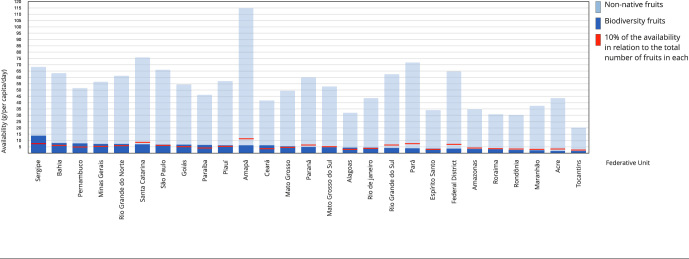
Availability of fruits in households in Brazilian Federative Units, 2017-2018.

Figure 1 shows the availability of biodiversity fruits in households in Brazilian states from 2017 to 2018. The average total availability (g/per capita/day) of fruits ranged from 114.89g to 20.55g. The states of Amapá and Tocantins had the highest and lowest participation rates, respectively. In 14 of the 27 Federative Units, the availability of biodiversity fruits was higher than 10% of the total fruits. Sergipe (13.64g), Bahia (8.08g), and Pernambuco (7.59g) had the greatest availability of biodiversity fruits, while Maranhão (2.21g), Acre (1.77g), and Tocantins (1.65g) had the lowest availability. Total and native fruit availability differed in some states, such as Sergipe (68.15g), which, even occupying the fourth position in the availability of total fruits, had the highest availability of native fruits (13.64g). In parallel, Amapá, with the highest total fruits (114.89g), had low acquisition of native fruits (6.05g).

**Figure 2:**
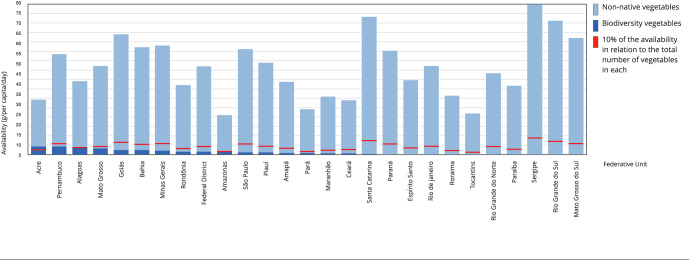
Availability of vegetables in households in Brazilian Federative Units, 2017-2018.

Figure 2 presents the absolute and relative availability of biodiversity vegetables in households in Brazilian states from 2017 to 2018. The average availability of vegetables (g/per capita/day) ranged from 79.99g to 20.69g. The states of Sergipe and Amazonas had the highest and lowest participation rates, respectively. Regarding biodiversity foods, Acre had the highest availability of vegetables (4.33g) and Espírito Santo and Paraná (0.2g), the lowest. Only two states had an amount equal to (Alagoas) or higher (Acre) than 10% of the total. On the other hand, eight states had no availability (Roraima, Rio de Janeiro, Tocantins, Rio Grande do Norte, Paraíba, Sergipe, Rio Grande do Sul, and Mato Grosso do Sul).

**Figure 3:**
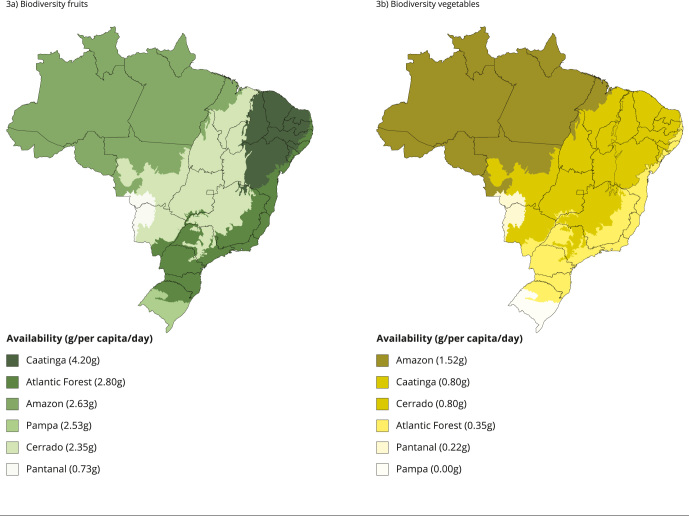
Availability of foods from Brazilian biodiversity in households according to biomes, 2017-2018.

Figure 3 presents the average availability of foods from Brazilian biodiversity in households according to biomes from 2017 to 2018. The availability of native fruits (Figure 3a) is low: the Caatinga occupies the first position (4.20g/per capita/day) while the Pantanal ranks last (0.73g/per capita/day). In the group of biodiversity vegetables (Figure 3b), the values are even lower, and the Amazon (1.52g) and the Pampa (0) occupy the first and last positions, respectively.

On the other hand, the availability of yerba mate in some Brazilian states was very high: the values in total grams (g/per capita/day) for Rio Grande do Sul, Santa Catarina, Paraná, and Mato Grosso do Sul, respectively, were 308.34g (21.5%), 164.4g (10.96%), 128.42g (9.11%) and 128.32g (11.17%) (data not shown).

## Discussion

The availability of foods from Brazilian biodiversity in households from 2017 to 2018, except for yerba mate, was low, especially in the vegetable group. In the analysis of consumption of native fruits by biomes, we observed that the Caatinga had the highest result while the Pantanal had the lowest. Regarding biodiversity vegetables, the Amazon and the Pampa had the highest and lowest results, respectively.

The low consumption of these foods can be mainly attributed to industrial food systems and global supply chains ^18^ that expand the production of products such as maize and soybeans. This undermines food diversity and contributes to further biodiversity loss. The standardization of food based on a few commodity-based products, without commitment to access to healthy food, leads to diseases and environmental crises ^19^.

Therefore, it is necessary to think about the consequences of this scenario for Brazilians, and for resilient production systems. Evidence shows that maize and soybeans are the basis of ultra-processed foods, which are harmful to health, increasing chronic noncommunicable diseases risk ^20^ ^,^ ^21^ ^,^ ^22^ and affecting the environment ^23^. Despite the omission of the topic, understanding that the loss of sociobiodiversity - the interrelationship between biological diversity and the diversity of sociocultural systems ^14^ - in Brazil is directly related to large-scale industrial production and the growing consumption of this type of food is essential. This has been causing a direct negative effect on the cultivation and consumption of plant food sources belonging to biodiversity systems, such as fruits and vegetables ^24^.

Data from the 2017-2018 POF show significant growth in the consumption of ultra-processed foods ^25^ while the price of this type of food tends to be lower, and even lower than that of fresh foods ^26^, resulting in increasingly limited access of the population to an adequate and healthy diet.

Studies highlight a continuous and growing prevalence of low fruit and vegetable consumption in different regions of Brazil, ranging from children ^27^ and adolescents ^28^ to adults ^29^ and older individuals ^30^. Moreover, the diversity of fruits consumed in households is low, with an average consumption of less than two fruits per day ^31^. Silva et al. ^32^ also warned of the low availability of regional foods from 2017 to 2018, when the caloric contribution was only 3.12% in the Brazilian diet. Despite the different focuses, studies with this approach have in common the evidence of the urgency to develop strategies to improve the promotion of healthier eating in the daily life of the Brazilian population based on food culture.

Improving diet quality also involves access to healthy and quality food. A study in Brazil ^33^ showed a higher concentration of greengory, street markets, with food coming directly from the producer, and butcher shops in central and especially urban areas, making access to these types of establishments difficult for the low-income population living in the periphery. At the same time, evidence shows a higher concentration of establishments with priority sales of ultra-processed foods in Brazil ^34^, in areas known as food swamps. Strategies such as encouraging small establishments to sell products in nature, dedicate public spaces to the creation of community gardens and promote the purchase of biodiversity foods for school meals can increase the availability of these foods.

Considering environmental aspects is also relevant, since the relationship between food and sustainability is closer than thought. The study by van Dikj et al. ^35^ assessed populations at risk of hunger in different scenarios, of which one was focused on sustainability, highlighting development that respects environmental limits, and shows that this population tends to decrease if the world follows a more sustainable path. Moreover, the studies by Garzillo et al. ^23^ ^,^ ^36^ warn of the carbon footprint of the Brazilian diet and state that a diet rich in fruits and vegetables is a viable solution to contain the significant increase in the planet’s average temperature, reducing negative environmental effects, besides being directly associated with the prevention or reduction in cases of chronic diseases and serious impacts on the health and quality of life of the Brazilian population.

Important national ^6^ ^,^ ^37^ ^,^ ^38^ ^,^ ^39^ and international ^40^ ^,^ ^41^ official documents encourage the consumption of biodiversity foods as health and culture promoters for the population. The golden rule of the food guide for the Brazilian population is that fresh and minimally processed foods are always preferable to ultra-processed foods ^37^. The Food and Agriculture Organization of the United Nations (FAO) considers the food groups analyzed in this study favorable to the health of the population and beneficial for sustainable food chains and reiterates the fundamental role of family farming in all these processes ^41^.

The low availability of native foods shown in this study highlights a worrying contrast between the official discourse and the priorities given by Brazil to its native foods. Jones et al. ^42^ pointed the need for improvements in the consumption and commitment to foods from Brazilian biodiversity. In this study, Brazil was considered one of the twelve countries that presented satisfactory averages in the status and action items. However, we observed a low level of commitment.

Considering that Brazil is one of the countries with the best average in terms of agrobiodiversity status - which refers to “*‘the variety and variability of animals, plants, and microorganisms that are used directly or indirectly for food and agriculture’, and is crucial for resilient and sustainable food systems*” ^24^ (p. 1) - and comparing these data with the numbers presented in this study, theory and practice have a clear counterpoint. In other words, being a country seen with good eyes for its biodiverse food system is not enough, since the availability of these foods in Brazilian households is significantly low. These numbers show the need to expand and qualify public and private actions to improve the commitment to the environmental and health agenda in the medium and long term.

The consumption of yerba mate, evaluated separately, is much higher than any other food, which shows that this product is very present in the food culture of the states of Mato Grosso do Sul, Rio Grande do Sul, Paraná, and Santa Catarina, where it is mainly consumed as mate, an infusion of yerba mate with hot water ^43^. This preparation has a strong cultural representation in terms of collective and shared consumption, which is very important, as the high consumption of this food preserves the food culture of these states and stimulates the local economy.

This study has limitations regarding the database. First, it considers only the names of the foods reported by the households and their variations and synonyms, since the POF data does not have the scientific nomenclature of these foods. Another limitation is that the database does not include food shared with people who are not part of the household or food waste. The data used also do not include food consumed outside the home, although this is not such a relevant limitation, since most food consumption in Brazil is still concentrated in the home, accounting for almost 85% of total energy consumption ^44^. Regarding biomes, another limitation is that the POF does not provide a more precise identification of the location of the households in each state, since the database omits specific information, such as address, telephone number, or number of the census sector where the household is located.

On the other hand, the biodiversity food classification used in this study helped understand which foods are currently purchased. Another strength is the POF food and beverage purchasing database, which, although it does not show actual food consumption but rather patterns of availability at the household level ^44^, has a high enough level of detail to identify the foods studied, beyond the scope and representativeness of the Brazilian population as a whole. Moreover, the division by approximate area of the biomes according to the IBGE map resulted in more detailed data. This is the first study to describe the household availability of foods from Brazilian biodiversity in all Brazilian states and biomes, highlighting the importance of focusing on this type of topic in research in the area of food.

## Conclusion

The narrative of a rich and diverse native food system with a globally recognized identity unfortunately does not match the availability of native species in Brazilian households, regardless of the biome or state analyzed. The results are unsatisfactory and far below what is expected from a rich territory with a native food system that stands out worldwide for its biodiversity. A greater commitment to the environment and a stronger call for actions that reinforce the consumption of biodiversity fruits and vegetables in daily life are essential. In this way, the global impression of a varied food biodiversity can be aligned with the practice of the food available on the Brazilian table.
